# Systematic mutation analysis of KIAA0767 and KIAA1646 in chromosome 22q-linked periodic catatonia

**DOI:** 10.1186/1471-244X-5-36

**Published:** 2005-10-14

**Authors:** Gerald Stöber, Bernd Kohlmann, Markus Siekiera, Claudia Rubie, Micha Gawlik, Kerstin Möller-Ehrlich, Thomas Meitinger, Thomas Bettecken

**Affiliations:** 1 Department of Psychiatry and Psychotherapy, University of Würzburg, Füchsleinstraße 15, 97080 Würzburg, Germany; 2 Department of Child and Youth Psychiatry and Psychotherapy, University of Würzburg, Füchsleinstraße 15, 97080 Würzburg, Germany; 3 Department of General, Vascular and Paediatric Surgery, University of the Saarland, Homburg/Saar 66421, Germany; 4 Institute of Human Genetics, Technical University of Munich & GSF Ingolstädter Landstr. 1, 85764 Neuherberg, Germany; 5 Max-Planck Institute of Psychiatry, Kraepelinstr. 2–10, 80804 Munich, Germany

## Abstract

**Background:**

Periodic catatonia is a familial subtype of schizophrenia characterized by hyperkinetic and akinetic episodes, followed by a catatonic residual syndrome. The phenotype has been evaluated in two independent genome-wide linkage scans with evidence for a major locus on chromosome 15q15, and a second independent locus on chromosome 22q_tel_.

**Methods:**

In the positional and brain-expressed candidate genes KIAA0767 and KIAA1646, we searched for variants in the complete exons and adjacent splice-junctions as well as in parts of the 5'- and 3'-untranslated regions by means of a systematic mutation screening in individuals from chromosome 22q-linked pedigrees.

**Results:**

The mutation scan revealed 24 single nucleotide polymorphisms, among them two rare codon variants (KIAA0767: S159I; KIAA1646: V338G). However, both were neither found segregating with the disease in the respective pedigree nor found at a significant frequency in a case-control association sample.

**Conclusion:**

Starting from linkage signals at chromosome22q_tel _in periodic catatonia, we screened two positional brain-expressed candidate genes for genetic variation. Our study excludes genetic variations in the coding and putative promoter regions of KIAA0767 and KIAA1646 as causative factors for periodic catatonia.

## Background

The phenotype of periodic catatonia is characterized by hyperkinetic and akinetic episodes with parakinetic movements in a bipolar course, accompanied by delusional or hallucinatory symptoms, and followed by residual states with psychomotor features [1,2]. The estimated lifetime prevalence of periodic catatonia is ~0.001 in the general population. Evidence for significant linkage to chromosome 15q15 was obtained and replicated in two independent genome-wide linkage scans [3,4]. Mainly supported by a single four-generation pedigree, a second locus was identified on chromosome 22q with a maximum multipoint LOD score of 2.59 (θ = 0.0) under an autosomal dominant model at marker *D22S1169*, and with a heterogeneity Z_max _of 1.57 with an estimated 38% of families linked [3]. Preliminary findings had suggested a link between *MLC1 *and catatonia via a putative dominantly acting missense mutation cosegregating in a large pedigree, but further analyses have excluded sequence variants of *MLC1 *as causing chromosome 22q-linked catatonia. MLC1 is the disease causing gene for autosomal recessively inherited megalencephalic leukoencephalopathy with subcortical cysts [5-7]. A systematic mutation screening in 140 index cases with periodic catatonia and five cases with MLC detected a high degree of sequence diversity of *MLC1 *with evidence for further allelic heterogeneity of *MLC1 *mutations in MLC, but unfortunately the study failed to validate an association of schizophrenia to genetic variants of *MLC1*. Among periodic catatonia index cases, the mutation scan revealed 15 different single nucleotide polymorphisms, among them three coding variants: two of them were observed in controls at a significant frequency, and the L309M variant, that was previously supposed to be the causative factor for chromosome 22q_tel _linked periodic catatonia, was found non-segregating in a further multiplex pedigree [6]. In addition, MLC1 is a 377 amino acid protein with preferential expression in the brain and peripheral white blood cells. In the brain, MLC1 is specifically expressed in distal astroglial processes in perivascular, subependymal, and subpial regions. Although MLC1 shares low homology with human voltage-gated potassium channels, addressing the membrane topology and cellular localization of MLC1 supports the possible transport function of MLC1 for a specific, yet unknown substrate [8,9]. Thus, mutations in *MLC1 *are causative for MLC, but can be excluded as a susceptibility factor in schizophrenia. Reports of other groups also failed to support an aetiological relevance of this gene in schizophrenia [10-12] and more importantly, these studies [6,12] rule out that spongiform leukodystrophies and subtypes of schizophrenia are allelic disorders.

Under the assumption of disease heterogeneity and allelic heterogeneity [13], we screened in a systematic approach the positional candidate genes KIAA1646 and KIAA0767, located between 45.34–45.45 Mb [14], for genetic variation. Human KIAA0767 is a mitochondrial protein of 578 amino acids (aa) with high expression in adult brain and strong pro-apoptotic effect [15,16]. KIAA1646 is a cytoplasmatic and membrane-associated ceramide kinase (CERK) of 537 amino acids [17]. CERK is acting in the signal transduction cascade and is suggested to be involved in the process of synaptic vehicle fusion [18]. KIAA0767 and KIAA1646 consist of 18 and 13 exons, respectively, spanning each ~50 kb on genomic DNA.

## Methods

We selected two affected individuals (933, 1045) from different branches of F20, and two cases (727, 857) of smaller pedigrees F15 and F17, which were compatible with linkage to chromosome 22q [19], as well as DNA of a healthy individual as control. In the association study we included 115 unrelated cases with periodic catatonia (66 males; mean age first hospitalisation: 26.2 years, SD 10.5; age at assessment: 44.6 years, SD 17.1) and 110 blood donors as controls (60 males; mean age at assessment: 29.5 years, SD 9.4). All subjects actively participated in the study after giving informed consent. The Ethics Committee of the University of Würzburg had approved the study.

Primers covered in overlapping fragments parts of the 5'-UTR containing putative promoter regions [20] and were allocated in intronic regions to encompass the complete exon and adjacent splice-junctions as well as parts of the 3'-UTR up to ~1.0 kb. PCR (30 sec at 94°C, 30 sec at 57°/60°C, and 30 sec at 72°C for 32 cycles) was carried out in 25 μl reaction volumes containing ~80 ng genomic DNA, 20 pmol of each primer, 200 μM of each dNTP, 0.5 U Taq DNA polymerase (Fermentas), and buffer as supplied by the manufacturer in a MJ Research 96-well block Tetrad thermocycler (Waltham, MA). Exon 1 of CERK was not sequenced because of technical difficulties. PCR products were purified by solid phase extraction and bidirectionally sequenced with ABI PRISM 3100 Genetic Analyzer (Applied Biosystems, Foster City, CA, USA) followed by computer-assisted analyses. For restriction fragment analyses (RFLP) PCR products were amplified on a Thermocycler (Biometra, Göttingen), and subsequently digested with the appropriate enzyme (CERK V338G: DraIII; KIAA0767 S159I: MboI). Fragments were resolved on a 10% PAA gel containing 1.0 × TBE at 15 V/cm^2 ^followed by silverstaining.

## Results

In the mutation screening sample we identified seven single nucleotide polymorphisms (SNPs) at the KIAA0767/DIP locus (Table 1). Nt 45379466 G>T changes a serine to isoleucine (S159I). We identified case 857 as a heterozygote carrier; the variant, however, did not co-segregate with the disease in the multiplex pedigree. The SNP was introduced through an unaffected individual, who married into the family. In addition, we did not observe 159I in a sample of 450 chromosomes (115 index cases with periodic catatonia and 110 controls, respectively). These findings coincidently indicated that 159I is a rare variant, but not associated with disease susceptibility.

**Table 1:** sequence variable at the KIAA0767/DIP locus on chromosome 22q13. 3

Nucleotide position *	DNA level	Nucleotide change	Codon	SNP-database	Genotypes of the individuals from each family					
					933	1045	727	857	Ctrl	Allele frequency

45377716	Intron 3	C>T	-	rs 2076708	CC	CC	CC	CC	CT	C: 0.9 T: 0.1
45379466	Exon 5	G>T	S1591	-	GG	GG	GG	GT	GG	G: 0.9 T: 0.1
45385184	Intron 11	C>T	-	-	CT	CC	CC	CC	CT	C: 0.8 T: 0.2
45385356	Intron 12	A>C	-	rs 2076711	AA	AA	AA	AA	AC	A: 0.9 C: 0.1
45391987	Intron 16	C>T	-	-	CC	CC	CC	CC	CT	C: 0.9 T: 0.1
45392051	Intron 16	T>C	-	-	TT	TT	TC	TC	TC	T: 0.7 C: 0.3
45392996	Intron 16	G>A	-	rs 2236028	GA	GG	GG	GG	GA	G: 0.8 A: 0.2

* nt position according the UCSC Genome Browser May 2004 assembly [14; NM\_022766].

Nucleotide changes found by automated sequencing of amplicons of four individuals from pedigrees segregating periodic catatonia evaluated in a genome-wide linkage scan [3, 19]: 933 and 1045 (F20), 727 and 857 (F15,F17), and a control subject.

The systematic scan of KIAA1646/CERK resulted in a total of 17 SNPs (Table 2). We observed the coding variant 338G by sequencing DNA-amplicons of a control subject. In a subsequent case-control association study, we found a frequency of the heterozygous genotype of 0.9% (2 out of 220 alleles) compared to a frequency of the heterozygous genotype of 2.2% in periodic catatonia (5 out of 230 alleles; ns). In addition, the variants C50 and D377 were both non-segregating within the multiplex pedigree F20, as did the synonymous N500 in the respective pedigrees.

**Table 2:** Polymorphisms at the KIAA1646/CERK locus in periodic catatonia

Nucleotide position*	DNA level	Nucleotide change	Codon	SNP-database	Genotypes of the individuals from each family					
					933	1045	727	857	Ctrl	Allele frequency

45437424	Exon 2	G>A	C50	rs 12166204	GG	GA	GG	GG	GG	G: 0.9; A: 0.1
45418024	Intron 7	C>T	-	rs 16995595	GT	CC	CC	CC	CC	C: 0.9; T: 0.1
45415928	Intron 7	C>G	-	-	CG	GG	CG	CC	CG	C: 0.5; G: 0.5
45415691	Intron 8	C>G	-	rs 5767329	CC	CC	CG	CG	CC	C: 0.8; G: 0.2
45411750	Intron 8	G>A	-	rs 9616098	GG	GG	GG	GG	GA	G: 0.9; A: 0.1
45411662	Exon 9	A>C	V338G	-	AA	AA	AA	AA	AC	A: 0.9; C: 0.1
45409793	Intron 10	G>A	-	-	GG	GG	GA	GA	GG	G: 0.8; A: 0.2
45408189	Exon 11	G>A	D377	-	GA	GG	GG	GG	GA	G: 0.8; A: 0.2
45406443	Exon 12	G>A	D502	-	GG	GG	GA	GA	GG	G: 0.8; A: 0.2
45403531	3'- UTR	T>A	-	-	TA	TT	TA	TA	AA	T: 0.5; A: 0.5
45403529	3'-UTR	T>C	-	rs 2542014	TC	TT	TC	TC	CC	T: 0.5; C: 0.5
45403096	3'-UTR	G>T	-	rs 8143065	GG	GG	GT	GT	GG	G: 0.8; T: 0.2
45402864	3'-UTR	C>T	-	rs 801719	CC	CC	CT	CT	CT	C: 0.7; T: 0.3
45402678	3'-UTR	C>A	-	rs 801720	CC	CC	CA	CA	AA	C: 0.6; A: 0.4
45402542	3'-UTR	G>A	-	rs 3747258	GG	GG	GA	GA	GG	G: 0.8; A: 0.2
45402502	3'-UTR	C>T	-	-	CC	CC	CT	CT	CC	C: 0.8; T: 0.2
45402374	3'-UTR	A>G	-	rs 2748348	AA	AA	AG	AG	AA	A: 0.8; G: 0.2

* nt position according the UCSC Genome Browser May 2004 assembly [14; NM\_022766].

Eleven of the 24 SNPs had not yet been deposited in current databases. At the CERK locus rs 2542014 was linked to a nearby (+2 bp) variant at nt 45403531. The intronic SNPs at nt 45415691C>G (KIAA1646) and nt 45392050 T>C (KIAA0767) were found at allelic frequencies useful for further LD-mapping studies.

## Discussion

Sustained interest in schizophrenia susceptibility on chromosome 22q is substantiated by several genome-wide linkage studies on schizophrenic psychoses [21-23]. Because of reports of schizophrenia susceptibility genes within the 22q11 region, this locus has obtained a high marker density compared to the 22q13 region [24]. Although only weak signals for linkage to chromosome 22q11 were received by recent multicenter and meta-analytic studies [25,26], subsets of pedigrees in the study by Mowry et al. [25] gave positive scores for chromosome 22q13, particularly if accounting for intersample heterogeneity. The pedigrees analyzed here were not part of these multicenter samples. We focused on the phenotype periodic catatonia [[27]; #605419] with a major disease locus at chromosome 15q15, and at least one further independent locus at chromosome 22q13, mainly supported by a large four-generational pedigree [3]. As it has been already demonstrated in several chromosome 15q15-linked pedigrees, haplotype analyses in pedigree F20 showed autosomal dominant transmission [3,6]. The lack of common haplotypes in unrelated families, however, added further evidence to genetic and allelic heterogeneity in periodic catatonia.

The chromosome 22q_tel _candidate locus of ~4 Mb comprises more than 45 genes [28], and harbours several genes involved in severe neuropsychiatric disorders, such as metachromatic leukodystrophy and megalencephalic leukoencephalopathy [[27]: #250100; #604004]. In a systematic mutation scan of positional candidates at the chromosome 22q_tel _candidate region we screened ~3400 nt of coding sequence including splice-donor sites and additionally parts of the 5'-UTR and 3'-UTR of KIAA0767 and KIAA1646, respectively. The mutation scan revealed 24 sequence variants, among them two rare codon variants (KIAA0767: S159I; KIAA1646: V338G). However, both neither were found to segregate with the disease in the respective pedigrees nor found at a significant frequency in periodic catatonia.

Human KIAA0767/DIP [GenBank: NM_015124; LocusLink: 23151] consists of 18 exons, spanning ~50 kb on genomic DNA [NT_011523.2]. KIAA0767 is a protein of 578 amino acids (aa) putatively localized in the mitochondrion [16]. KIAA0767 expression resulted in a significant loss of cell viability independent of the p53 status, and thus, termed death-inducing-protein (DIP) because of its strong pro-apoptotic effect [16]. DIP shows at least two transmembrane domains and multiple putative phosphorylation and glycolisation sites. The variant S159I, however, seems not to affect any of the various functional DIP/KIAA0767 protein domains.

Human KIAA1646/CERK [GenBank: NM_022766; LocusLink: 64781] consists of 13 exons, spanning ~50 kb on genomic DNA. CERK is a cytoplasmatic and membrane-associated protein of 537 amino acids with a calculated molecular weight of 60 kDa. It is a member of a new class of lipid kinases and acts in the signal transduction cascade of the control of apoptosis catalyzing specifically the phosphorylation of ceramide [17]. The component ceramide is thought to regulate apoptotic responses to stress, particularly those initiated by the mitochondria. CERK is highly expressed in brain and leukocytes, and is suggested to be involved in synaptic neurotransmitter release [18] or in phagocytosis and vehicle fusion in neutrophils and mast cells [17,29,30]. In CERK, the pleckstrin homology domain and the diacylglycerol kinase (DGK) catalytic domain were found non-polymorphic, but V338G was located in the central homologous region, near the casein kinase II phosphorylation site at S340, and V338 is conserved between human and mouse CERK [17,31]. Although V338G may be of functional relevance, it was not found associated with periodic catatonia. Recent reports that mutations in the related gene CERKL on chromosome 2q31 cause autosomal recessive retinitis pigmentosa (RP26) indicate a link of the ceramide kinase gene family to retinal neurodegeneration [32].

Starting from linkage signals at chromosome22q_tel _in periodic catatonia, we screened two positional brain-expressed candidate genes for genetic variation (KIAA0767/DIP; KIAA1646/CERK). Our study excludes variants at coding and putative promoter regions as causative factors in periodic catatonia, but do not exclude the involvement of other regulatory elements in intronic or extended promoter regions. Although negative, the present study narrowed down the putative susceptibility region, and provides a systematic SNP generation for forthcoming LD studies.

## Competing interests

The author(s) declare that they have no competing interests.

## Authors' contributions

BK, MS, CR performed laboratory assays, MG, KME performed the data-analysis and drafted the manuscript, TM, TB participated in the design of the study and its coordination, GS participated in the design of the study, interpretation of the data, and drafting of the manuscript. All authors read and approved the final manuscript.

## Pre-publication history

The pre-publication history for this paper can be accessed here:


